# Blow-up analysis for a periodic two-component *μ*-Hunter–Saxton system

**DOI:** 10.1186/s13660-018-1903-8

**Published:** 2018-11-12

**Authors:** Yunxi Guo, Tingjian Xiong

**Affiliations:** 1 School of Mathematics, Zunyi Normal University, Zunyi, China; 2 Department of Applied Mathematics, Sichuan University of Science and Engineering, Zigong, China

**Keywords:** 35D05, 35G25, 35L05, 35Q35, Two-component *μ*-Hunter–Saxton system, Wave-breaking criteria, Blow-up phenomena

## Abstract

The two-component *μ*-Hunter–Saxton system is considered in the spatially periodic setting. Firstly, two wave-breaking criteria are derived by employing the transport equation theory and the localization analysis method. Secondly, a sufficient condition of the blow-up solutions is established by using the classic method. The results obtained in this paper are new and different from those in previous works.

## Introduction

In this article, we consider the periodic two-component *μ*-Hunter–Saxton system derived by Zuo [1]

1 $$ \textstyle\begin{cases} \mu (u_{t})-u_{txx}=2\mu (u)u_{x}-2\sigma u_{x}u_{xx}-\sigma uu_{xxx}+ \rho \rho_{x}-\gamma_{1}u_{xxx},\quad t>0, x\in \mathbb{R}, \\ \rho_{t}=(u\rho )_{x}+2\gamma_{2}\rho_{x}, \quad t>0, x\in \mathbb{R}, \\ u(0,x)=u_{0}(x), \quad x\in \mathbb{R}, \\ \rho (0,x)=\rho_{0}(x), \quad x\in \mathbb{R}, \\ u(t,x+1)=u(t,x), \quad t>0, x\in \mathbb{R}, \\ \rho (t,x+1)=\rho (t,x), \quad t>0, x\in \mathbb{R}, \end{cases} $$

where $u(t,x)$ and $\rho (t,x)$ are time-dependent functions on the unit circle $\mathbb{S}=\mathbb{R}/\mathbb{Z}$, the real dimensionless constant $\sigma \in \mathbb{R}$ is a parameter which provides the competition, or balance, in fluid convection between nonlinear steepening and amplification due to stretching. $\mu (u)=\int_{\mathbb{S}}u\,dx$ denotes its mean and $\gamma_{i}\in \mathbb{R}$, $i=1,2$. It is shown in [1] that system (1) is an Euler equation with bi-Hamilton structure

$$\Gamma_{1}=\left(\begin{array}{cc} \partial_{x}A & 0\\ 0 & \partial_{x} \end{array}\right),\;\;\Gamma_{2}=\left(\begin{array}{cc} A(u)\partial_{x}+\partial_{x}A(u)-\gamma_{1}\partial_{x}^{3} & \rho \partial_{x}\\ \partial_{x}\rho & 2\gamma_{2}\partial_{x} \end{array}\right),$$

where $A(u)=\mu (u)-u_{xx}$, and it is also viewed as a bi-variational equation. Moreover, for $\gamma_{i}=0$, $i=1,2$, system (1) has a Lax pair given by

$$\begin{aligned} \psi_{xx}=\lambda \bigl(A(u)-\lambda^{2}\rho^{2} \bigr)\psi ,\quad\quad \psi_{t}=\biggl(u-\frac{1}{2 \lambda }\biggr) \psi_{x}-\frac{1}{2}u_{x}\psi , \end{aligned}$$

where *λ* is a spectral parameter (see [1]). Recently, Liu and Yin [2, 3] investigated the Cauchy problem for system (1). In [2], the local well-posedness and several precise blow-up criteria for the system were obtained. Under the conditions $\mu_{0}=0$ and $\mu_{0}\neq 0$, the sufficient conditions of blow-up solutions were presented. The global existence for strong solution for system (1) in the Sobolev space $H^{s}(\mathbb{S}) \times H^{s-1}(\mathbb{S})$ with $s=2$ is also given [2], and in [3], the existence of global weak solution is established for the periodic two-component *μ*-Hunter–Saxton system. The objective of the present paper is to focus mainly on wave-breaking criterion and several sufficient conditions of blow-up solutions.

If *t* is replaced by −*t*, and $\gamma_{i}=0$ ($i=1,2$) in system (1), in fact, system (1) has significant relationship with several models describing the motion of waves at the free surface of shallow water under the influence of gravity. Such as *μ*-Camassa–Holm equation [4–6], *μ*–*b* equation [7], two-component periodic Hunter–Saxton system [8–12], and two-component Dullin–Gottwald–Holm system [13, 14]. If $\mu -\partial^{2}_{x}$ and *t* are replaced by $1-\partial^{2}_{x}$ and −*t* in system (1) respectively, system (1)($\gamma_{i}=0$ ($i=1,2$)) becomes the two-component Camassa–Holm system, and its dynamic properties can be found in [15–24] and the references therein.

Integrating the first equation of system (1) over the circle $\mathbb{S}=\mathbb{R}/\mathbb{Z}$ and noting the periodicity of *u*, we have $\mu (u_{t})=0$. Making use of system (1), we have that $\int_{\mathbb{S}}(u^{2}_{x}+\rho^{2})\,dx$ is conserved in time. In what follows we denote

2 $$\begin{aligned} \mu_{0}=\mu (u_{0})=\mu (u)= \int_{\mathbb{S}}u(t,x)\,dx \end{aligned}$$

and

3 $$\begin{aligned} \mu_{1}= \biggl( \int_{\mathbb{S}}u^{2}_{x}(t,x)+ \rho^{2}(t,x)\,dx \biggr)^{ \frac{1}{2}}= \biggl( \int_{\mathbb{S}}u^{2}_{x}(0,x) + \rho^{2}(0,x)\,dx \biggr)^{\frac{1}{2}}. \end{aligned}$$

Then $\mu_{0}$ and $\mu_{1}$ are constants independent of time *t*.

Notice that $\int_{\mathbb{S}}(u(t,x)-\mu_{0})\,dx=\mu_{0}-\mu_{0}=0$. From Remark 2.1 in [2], we get

4 $$\begin{aligned} \max_{x\in \mathbb{S}}\bigl[u(t,x)-\mu_{0}\bigr]^{2}& \leq \frac{1}{12} \int_{\mathbb{S}}u^{2}_{x}(t,x)\,dx \leq \frac{1}{12} \int_{\mathbb{S}}u ^{2}_{x}(t,x)+ \rho^{2}(t,x)\,dx \\ &=\frac{1}{12} \int_{\mathbb{S}}u^{2}_{x}(0,x) + \rho^{2}(0,x)\,dx= \frac{1}{12}\mu^{2}_{1}, \end{aligned}$$

which implies that the amplitude of wave remains bounded in any time. Namely, we have

5 $$\begin{aligned} \bigl\Vert u(t,\cdot ) \bigr\Vert _{L^{\infty }(\mathbb{S})}- \vert \mu_{0} \vert \leq \bigl\Vert u(t,\cdot )-\mu_{0} \bigr\Vert _{L^{\infty }(\mathbb{S})} \leq \frac{\sqrt{3}}{6}\mu_{1}, \end{aligned}$$

which results in

6 $$\begin{aligned} \bigl\Vert u(t,\cdot ) \bigr\Vert _{L^{\infty }(\mathbb{S})} \leq \vert \mu _{0} \vert +\frac{\sqrt{3}}{6}\mu_{1}. \end{aligned}$$

In fact, the initial-value problem (1) can be recast in the following:

7 $$ \textstyle\begin{cases} u_{t}-(\sigma u+\gamma_{1})u_{x}=A^{-1}\partial_{x}(2\mu_{0} u+\frac{ \sigma }{2}u^{2}_{x}+\frac{1}{2}\rho^{2}), \quad t>0, x\in \mathbb{R}, \\ \rho_{t}-(u+2\gamma_{2})\rho_{x}=\rho u_{x}, \quad t>0, x\in \mathbb{R}, \\ u(0,x)=u_{0}(x), \quad x\in \mathbb{R}, \\ \rho (0,x)=\rho_{0}(x), \quad x\in \mathbb{R}, \\ u(t,x+1)=u(t,x), \quad t>0, x\in \mathbb{R}, \\ \rho (t,x+1)=\rho (t,x), \quad t>0, x\in \mathbb{R}, \end{cases} $$

where $A=\mu -\partial^{2}_{x}$ is an isomorphism between $H^{s}$ and $H^{s-2}$ with the inverse $\nu =A^{-1}\omega $ given explicitly by

8 $$\begin{aligned} \nu (x)&=\biggl(\frac{x^{2}}{2}-\frac{x}{2}+\frac{13}{12}\biggr) \mu (\omega )+\biggl(x- \frac{1}{2}\biggr) \int_{0}^{1} \int^{y}_{0}\omega (s)\,ds \,dy \\ &\quad {} - \int_{0}^{x} \int^{y}_{0}\omega (s)\,ds \,dy + \int_{0}^{1} \int ^{y}_{0} \int^{s}_{0}\omega (r)\,dr \,ds \,dy. \end{aligned}$$

Commuting $A^{-1}$ and $\partial_{x}$, we get

9 $$\begin{aligned} A^{-1}\partial_{x}\omega (x)=\biggl(x-\frac{1}{2} \biggr) \int^{1}_{0}\omega (x)- \int^{x}_{0}\omega (y)\,dy + \int_{0}^{1} \int^{x}_{0}\omega (y)\,dy \,dx \end{aligned}$$

and

10 $$\begin{aligned} A^{-1}\partial^{2}_{x}\omega (x)=-\omega (x)+ \int^{1}_{0}\omega (x)\,dx . \end{aligned}$$

Note that if $f\in L^{2}(\mathbb{S})$, we have $A^{-1}f=(\mu -\partial ^{2}_{x})^{-1}f=g\ast f$, where we denote by ∗ convolution and *g* is the Green’s function of the operator $A^{-1}$ given by

11 $$\begin{aligned} g(x)=\frac{1}{2}\biggl(x-\frac{1}{2}\biggr)^{2}+ \frac{23}{24}, \end{aligned}$$

and the derivative of *g* can be assigned

12 $$ g_{x}(x)= \textstyle\begin{cases} 0, & x=0, \\ x-\frac{1}{2},&x\in (0,1). \end{cases} $$

The objective of the present paper is to focus mainly on a wave-breaking criterion and wave-breaking phenomena for system (1). The local well-posedness of system (1) is firstly established by using Kato’s theory. Then we present two wave-breaking criteria (see Theorem 3.1 and Theorem 3.2) and a wave-breaking phenomenon (Theorem 4.1) for system (1) in the Sobolev space $H^{s}(\mathbb{S})\times H^{s-1}( \mathbb{S})$ with $s\geq 2$. The results obtained in this paper are new and different from those in Liu and Yin’s work [2].

The rest of this paper is organized as follows. Section 2 states local well-posedness for the periodic two-component *μ*-Hunter–Saxton system. In Sect. 3, we employ the transport equation theory to prove a wave-breaking criterion in the Sobolev space $H^{s}(\mathbb{S}) \times H^{s-1}(\mathbb{S})$ with $s\geq 2$. An improved wave-breaking criterion also is presented in Sect. 3. Section 4 is devoted to the study of a wave-breaking phenomenon.

## Local well-posedness

In this section, we will establish the local well-posedness for system (1) by Kato’s theorem. For convenience, we present here Kato’s theorem. Consider the abstract quasilinear evolution equation

13 $$ \textstyle\begin{cases} \frac{dv}{dt}+A(v)v=f(v),\quad t\geq 0, \\ v(0)=v_{0}. \end{cases} $$

Let *X* and *Y* be two Hilbert spaces such that *Y* is continuously and densely embedded in *X*. Let $Q:Y\rightarrow X$ be a topological isomorphism, and let $\Vert \cdot \Vert _{X}$ and $\Vert \cdot \Vert _{Y}$ be the norms of the Banach spaces *X* and *Y*, respectively. Let $L(Y,X)$ denote the space of all bounded linear operators from *Y* to *X*. In particular, it is denoted by $L(X)$ if $X=Y$. If *A* is an unbounded operator, we denote the domain of *A* by $D(A)$. $[A,B]$ denotes the commutator of two linear operators *A* and *B*. The linear operator *A* belongs to $G(X,1,\beta )$, where *β* is a real number, if −*A* generates a $C_{0}$-semigroup such that $\Vert e^{-sA}\Vert _{L(X)}\leq e^{\beta s}$. The inner product in $H^{s}$ is denoted by $\langle \cdot ,\cdot \rangle_{s}$, particularly the $L^{2}$ inner product is $\langle \cdot ,\cdot \rangle $.

We make the following assumptions, where $\mu_{1}$, $\mu_{2}$, $\mu_{3}$, and $\mu_{4}$ are constants depending only on $\max \{ \Vert z\Vert _{Y}, \Vert y\Vert _{Y}\}$.

(I) $A(y)\in L(Y,X)$ for $y\in X$ with
  $$\begin{aligned} \bigl\Vert \bigl(A(y)-A(z)\bigr)w \bigr\Vert _{X}\leq \mu_{1} \Vert y-z \Vert _{X} \Vert w \Vert _{Y},\quad y,z,w\in Y \end{aligned}$$
  and $A(y)\in G(X,1,\beta )$ (i.e., $A(y)$ is quasi-m-accretive), uniformly on bounded sets in *Y*.
(II) $QA(y)Q^{-1}=A(y)+B(y)$, where $B(y)\in L(X)$ is bounded uniformly on bounded sets in *Y*. Moreover,
  $$\begin{aligned} \bigl\Vert \bigl(B(y)-B(z)\bigr)w \bigr\Vert _{X}\leq \mu_{2} \Vert y-z \Vert _{Y} \Vert w \Vert _{X},\quad y,z\in Y, w\in X. \end{aligned}$$
(III) $f: Y\rightarrow Y$ extends to a map from *X* into *X*, is bounded on bounded sets in *Y*, and satisfies
  $$\begin{aligned} \bigl\Vert f(y)-f(z) \bigr\Vert _{Y}\leq \mu_{3} \Vert y-z \Vert _{Y},\quad y,z\in Y \end{aligned}$$
  and
  $$\begin{aligned} \bigl\Vert f(y)-f(z) \bigr\Vert _{X}\leq \mu_{4} \Vert y-z \Vert _{X},\quad y,z\in Y. \end{aligned}$$

### Kato’s Theorem

([25])

*Assume that conditions* (I), (II), *and* (III) *hold*. *Given* $v_{0}\in Y$, *there is a maximal* $T>0$ *depending only on* $\Vert v_{0}\Vert _{Y}$ *and a unique solution* *v* *to system* (13) *such that*

$$\begin{aligned} v=v(\cdot , v_{0})\in C\bigl([0,T\bigr); Y)\cap C^{1} \bigl([0,T\bigr); X). \end{aligned}$$

*Moreover*, *the map* $v_{0}\longmapsto v(\cdot , v_{0})$ *is a continuous map from* *Y* *to* $C([0,T); Y)\cap C^{1}([0,T); X)$.

Set

$$f(z)=\left(\begin{array}{c} \partial_{x}{\left(\mu -\partial_{x}^{2}\right)}^{-1}(2\mu_{0}u+\frac{\sigma }{2}u_{x}^{2}+\frac{1}{2}\rho^{2})\\ u_{x}\rho \end{array}\right),$$

$Y=H^{s}(\mathbb{S})\times H^{s-1}(\mathbb{S})$, $X=H^{s-1}( \mathbb{S})\times H^{s-2}(\mathbb{S})$, $\varLambda =(1-\partial^{2}_{x})^{ \frac{1}{2}}$, $\varLambda_{\mu }=(\mu -\partial^{2}_{x})^{\frac{1}{2}}$,

$$z=\left(\begin{array}{c} u\\ \rho \end{array}\right)\;\text{and}\;Q=\left(\begin{array}{cc} \Lambda_{\mu } & 0\\ 0 & \Lambda_{\mu } \end{array}\right).$$

Obviously, *Q* is an isomorphism of $H^{s}\times H^{s-1}$ onto $H^{s-1}\times H^{s-2}$.

The local well-posedness for system (1) is collected in the following.

### Theorem 2.1

*Given* $z_{0}=(u_{0},\rho_{0})\in H^{s}( \mathbb{S})\times H^{s-1}(\mathbb{S})$, $s\geq 2$, *then there exists a maximal* $T=T(\Vert z_{0} \Vert _{H^{s}(\mathbb{S})\times H^{s-1}(\mathbb{S})})>0$ *and a unique solution* $z=(u,\rho )$ *to system* (1) *such that*

$$ z=z(\cdot ,z_{0})\in C\bigl([0,T\bigr);H^{s}(\mathbb{S})) \cap C^{1}\bigl([0,T\bigr);H ^{s-1}(\mathbb{S})). $$

### Proof

Since there are some similarities with the proof of Theorem 3.1 in [14], here we omit the proof of the theorem. □

## Wave-breaking criteria

### Lemma 3.1

([26])

*Let* $T>0$ *and* $v\in C^{1}([0,T); H^{2}(R))$. *Then*, *for every* $t\in [0,T)$, *there exists at least one point* $\xi (t)\in R$ *with*

$$ m(t):=\inf_{x\in R}\bigl(v_{x}(t,x) \bigr)=v_{x}\bigl(t,\xi (t)\bigr). $$

*The function* $m(t)$ *is absolutely continuous on* $(0,T)$ *with*

$$ \frac{dm(t)}{dt}=v_{tx}\bigl(t,\xi (t)\bigr)\quad \textit{a.e. on } (0,T). $$

Now, consider the initial value problem for the Lagrangian flow map:

14 $$ \textstyle\begin{cases} q_{t}=u(t,-q)+2\gamma_{2},\quad t\in [0,T), \\ q(0,x)=x, \quad x\in \mathbb{R}, \end{cases} $$

where *u* denotes the first component of the solution $z=(u,\rho )$ to system (1). Applying classical results from ordinary differential equations, one can obtain the result.

### Lemma 3.2

*Let* $u\in C([0,T);H^{s}(\mathbb{R}))\cap C^{1}([0,T);H^{s-1}(\mathbb{R}))$, $s\geq 2$. *Then Eq*. (14) *has a unique solution* $q\in C^{1}([0,T)\times \mathbb{R};\mathbb{R})$. *Moreover*, *the map* $q(t,\cdot )$ *is an increasing diffeomorphism of* $\mathbb{R}$ *with*

15 $$\begin{aligned} q_{x}(t,x)=\exp \biggl(- \int^{t}_{0}u_{x}\bigl(s,-q(s,x)\bigr) \,ds\biggr)>0,\quad (t,x) \in [0,T)\times \mathbb{R}. \end{aligned}$$

### Lemma 3.3

*Let* $z_{0}=(u_{0},\rho_{0})\in H^{s}( \mathbb{S})\times H^{s-1}(\mathbb{S})$, $s\geq 2$, *and let* $T>0$ *be the maximal existence time of the corresponding solution* $z=(u,\rho )$ *to system* (1). *Then it has*

16 $$\begin{aligned} \rho \bigl(t,-q(t,x)\bigr)q_{x}(t,x)=\rho_{0}(-x), \quad (t,x)\in [0,T)\times \mathbb{R}. \end{aligned}$$

### Theorem 3.1

*Let* $z_{0}=(u_{0},\rho_{0})\in H^{s}( \mathbb{S})\times H^{s-1}(\mathbb{S})$ *with* $s\geq 2$, *and* $z=(u, \rho )$ *be the corresponding solution to* (1). *Assume that* $T>0$ *is the maximal existence time*. *Then*

17 $$\begin{aligned} T< \infty \quad \Rightarrow\quad \int^{T}_{0} \Vert u_{x} \Vert _{L^{\infty }(\mathbb{S})}\,d\tau =\infty . \end{aligned}$$

### Proof

Since the two equations for *u* and *ρ* in system (7) satisfy the transport structure

$$\begin{aligned} \partial_{t}f+v\partial_{x}f=F. \end{aligned}$$

Therefore, we can complete the proof of Theorem 3.1 by making use of conservation laws and the localization analysis in transport equation theory (see Theorems 3.1 and 3.2) in [18]. The detailed proof can be found in [18]. □

### Theorem 3.2

*Let* $(u_{0}, \rho_{0})\in H^{s}\times H ^{s-1} $ *with* $s > 3/2$, *and* $T > 0$ *be the maximal time of existence of the solution* $(u, \rho )$ *to system* (1) *with initial data* $(u_{0}, \rho_{0})$. *Then the corresponding solution* $(u, \rho )$ *blows up in finite time* $T < \infty $ *if and only if*

18 $$\begin{aligned} \lim_{t\rightarrow T}\Bigl\{ \sup_{x\in \mathbb{S}} \vert u_{x} \vert \Bigr\} =+ \infty . \end{aligned}$$

*Furthermore*, *if* $\sigma \geq 1$, *then the corresponding solution* $(u, \rho )$ *blows up in finite time* $T < \infty $ *if and only if*

19 $$\begin{aligned} \lim_{t\rightarrow T^{-}}\biggl\{ \sup_{x\in \mathbb{S}} \sqrt{ \frac{ \sigma }{2}}u_{x}\biggr\} =+\infty . \end{aligned}$$

### Proof

By Theorem 2.1 and a simple density argument, we need only to prove this theorem for $s \geq 3$. We may also assume $u_{0}\neq 0$, otherwise it is trivial. Let $T > 0$ be the maximal time of existence of the corresponding solution $(u, \rho )$ to system (1). We first prove the case in (18). Assume that $T < \infty $ and (18) is not true. Then there is some positive number $\varOmega > 0$ such that

20 $$\begin{aligned} \vert u_{x} \vert \leq \varOmega , \quad \forall (t, x) \in [0, T) \times R. \end{aligned}$$

Therefore, Theorem 3.1 implies that the maximal existence time $T = \infty $, which contradicts the assumption that $T < \infty $.

Now, we try to prove the blow-up criterion (19). Since $\sup_{x\in \mathbb{S}}(v_{x}(t,x))=-\inf_{x\in \mathbb{S}}(-v_{x}(t, x))$, we define

21 $$\begin{aligned} N(t)=u_{x}\bigl(t,-\xi (t)\bigr)=\sup_{x\in \mathbb{S}}u_{x}(t,x), \quad t \in [0, T). \end{aligned}$$

Obviously,

22 $$\begin{aligned} u_{xx}\bigl(t,-\xi (t)\bigr)=0, \quad t \in [0, T). \end{aligned}$$

Since $q(t,\cdot )$ defined by (14) is a diffeomorphism of the circle for any $t \in [0,T )$, there exists $x_{1}(t) \in \mathbb{S}$ such that

23 $$\begin{aligned} q\bigl(t,x_{1}(t)\bigr)=\xi (t), \quad t \in [0, T). \end{aligned}$$

Along the trajectory of $q(t,x_{1}(t))$, we have

24 $$\begin{aligned} \frac{d\rho (t,-\xi (t))}{dt}&=\frac{d\rho (t,-q(t,x_{1}(t)))}{dt}= \rho \bigl(t,-q(t,x_{1}) \bigr)u_{x}\bigl(t,-q(t,x_{1})\bigr) \\ & =\rho \bigl(t,-\xi (t)\bigr) u_{x}\bigl(t,-\xi (t)\bigr), \quad t \in [0, T). \end{aligned}$$

Differentiating the first equation of system (7) and using the equality $\partial^{2}_{x}\varLambda^{-2}_{\mu } f=-f+\int^{1}_{0}f\,dx$, we have

25 $$\begin{aligned} u_{tx}-(\sigma u+\gamma_{1})u_{xx}= \frac{\sigma }{2}u^{2}_{x}-2\mu _{0}u- \frac{1}{2}\rho^{2}+2\mu^{2}_{0}+ \int_{0}^{1}\biggl(\frac{\sigma }{2}u ^{2}_{x} +\frac{1}{2}\rho^{2}\biggr)\,dx. \end{aligned}$$

Along the trajectory of $q(t,x_{1}(t))$, (25) can be rewritten as the following form:

26 $$\begin{aligned} N'(t)=\frac{\sigma }{2}N^{2}(t)-\frac{1}{2} \rho^{2}(t)+d\bigl(t,-\xi (t)\bigr), \end{aligned}$$

where ′ denotes the derivative with respect to *t* and $d(t)=-2\mu _{0}u+2\mu^{2}_{0}+\int_{0}^{1}(\frac{\sigma }{2}u^{2}_{x}+ \frac{1}{2}\rho^{2})\,dx$.

Assume that (19) is not valid, then there is some positive number *Ω* such that

27 $$\begin{aligned} \sup_{x\in \mathbb{S}} \sqrt{\frac{\sigma }{2}}u_{x}\leq \varOmega , \quad \forall (t,x)\in [0,T)\times \mathbb{S}, \end{aligned}$$

then, from (24), for each $x\in \mathbb{S}$, we get

28 $$\begin{aligned} \bigl\vert \rho \bigl(t,-\xi (t)\bigr) \bigr\vert = \bigl\vert \rho (0) \bigr\vert e^{\int^{t}_{0} u_{x}\,dt} \leq \Vert \rho_{0} \Vert _{L^{\infty }}e^{\sqrt{\frac{2}{ \sigma }} \varOmega t}, \end{aligned}$$

from which we obtain

29 $$\begin{aligned} \bigl\Vert \rho \bigl(t,-\xi (t)\bigr) \bigr\Vert _{L^{\infty }} \leq \Vert \rho _{0} \Vert _{L^{\infty }}e^{\sqrt{\frac{2}{\sigma }}\varOmega t}. \end{aligned}$$

In order to proceed with the proof, next we need to obtain the lower bound of $d(t,-\xi (t))$.

30 $$\begin{aligned} d\bigl(t,-\xi (t)\bigr) =&-2\mu_{0}u+2\mu^{2}_{0} + \int^{1}_{0} \frac{\sigma }{2}u_{x}^{2}+ \frac{1}{2}\rho^{2}\,dx \\ \geq& -2 \vert \mu_{0}u \vert +2\mu^{2}_{0}+ \frac{1}{2}\mu^{2}_{1} \\ \geq &-\frac{\sqrt{3}}{3} \vert \mu_{0} \vert \mu_{1}+\frac{1}{2} \mu^{2}_{1}. \end{aligned}$$

Let

31 $$\begin{aligned} Q(t)&=\sqrt{\frac{\sigma }{2}}N(t)+\sqrt{\frac{\sigma }{2}} \Vert u_{0,x} \Vert _{L^{\infty }}+\mu_{1}+\sqrt{ \frac{ \sqrt{3}}{3} \vert \mu_{0} \vert \mu_{1}} \\ &\quad{} +\frac{\sqrt{2}}{2} \Vert \rho_{0} \Vert _{L^{\infty }}e ^{\sqrt{\frac{2}{\sigma }}\varOmega t}, \end{aligned}$$

then

32 $$\begin{aligned} Q(0)&=\sqrt{\frac{\sigma }{2}}N(0)+\sqrt{\frac{\sigma }{2}} \Vert u_{0,x} \Vert _{L^{\infty }}+\mu_{1}+\sqrt{ \frac{\sqrt{3}}{3} \vert \mu_{0} \vert \mu_{1}} \\ &\quad{} +\frac{\sqrt{2}}{2} \Vert \rho_{0} \Vert _{L^{\infty }}>0. \end{aligned}$$

We now claim that

33 $$\begin{aligned} Q(t)>0,\quad t\in [0,T). \end{aligned}$$

Assume the contrary that there is $t_{0}\in [0,T)$ such that $Q(t_{0})<0$. Let $t_{1}=\max \{t< t_{0}; Q(t)=0\}$. Then $Q(t_{1})=0$ and $Q'(t_{1})<0$, namely

34 $$\begin{aligned} &\sqrt{\frac{\sigma }{2}}N(t_{1}) =-\sqrt{\frac{\sigma }{2}} \Vert u_{0,x} \Vert _{L^{\infty }}- \mu_{1}-\sqrt{\frac{\sqrt{3}}{3} \vert \mu_{0} \vert \mu_{1}} -\frac{ \sqrt{2}}{2} \Vert \rho_{0} \Vert _{L^{\infty }}e^{\sqrt{\frac{2}{ \sigma }}\varOmega t_{1}} \end{aligned}$$

and

35 $$\begin{aligned} \sqrt{\frac{\sigma }{2}}N'(t_{1}) < -\sqrt{ \frac{1}{\sigma }} \varOmega \Vert \rho_{0} \Vert _{L^{\infty }}e^{\sqrt{\frac{2}{ \sigma }} \varOmega t_{1}}< 0. \end{aligned}$$

Recalling (26) and using (33), we have

36 $$\begin{aligned} N'(t_{1}) & =\frac{\sigma }{2}N^{2}(t_{1})-\frac{1}{2} \rho^{2}(t_{1})+d(t_{1}) \\ & > \biggl( -\sqrt{\frac{\sigma }{2}} \Vert u_{0,x} \Vert _{L^{\infty }}-\mu_{1}-\sqrt{\frac{\sqrt{3}}{3} \vert \mu _{0} \vert \mu_{1}} -\frac{\sqrt{2}}{2} \Vert \rho_{0} \Vert _{L^{\infty }}e^{\sqrt{\frac{2}{\sigma }}\varOmega t_{1}} \biggr)^{2} \\ & \quad {} + \biggl[ -\frac{\sqrt{3}}{3} \vert \mu_{0} \vert \mu_{1}+ \frac{1}{2}\mu^{2}_{1} \biggr]- \frac{1}{2} \Vert \rho_{0} \Vert ^{2}_{L^{\infty }}e^{2\sqrt{\frac{2}{\sigma }}\varOmega t_{1}} \\ & >0, \end{aligned}$$

which is a contradiction to (35). This verifies that (33) is valid. Therefore, choosing arbitrary $x\in \mathbb{S}$, we have

37 $$\begin{aligned} &\sup_{x\in R}\sqrt{\frac{\sigma }{2}}u_{x}(t,x) \\ &\quad \geq -\sqrt{\frac{\sigma }{2}} \Vert u_{0,x} \Vert _{L^{ \infty }}-\mu_{1}-\sqrt{\frac{\sqrt{3}}{3} \vert \mu_{0} \vert \mu _{1}} -\frac{\sqrt{2}}{2} \Vert \rho_{0} \Vert _{L^{\infty }}e ^{\sqrt{\frac{2}{\sigma }}\varOmega t}, \end{aligned}$$

recalling the assumption

$$\begin{aligned} \sup_{x\in R} \sqrt{\frac{\sigma }{2}}u_{x}\leq \varOmega ,\quad \forall (t,x)\in [0,T)\times R, \end{aligned}$$

we get $\sqrt{\frac{\sigma }{2}}\vert u_{x}\vert <+\infty $. This contradicts our assumption $T<\infty $, which completes the proof of Theorem 3.2. □

## Wave-breaking phenomenon

In this section, we give a new blow-up phenomenon. To prove the blow-up phenomenon, the following lemma is crucial.

### Lemma 4.1

([26])

*Let* *g* *be a monotone function on* $[a,b]$ *and* *f* *be a real continuous function on* $[a,b]$. *Then there exists* $\xi \in [a,b]$ *such that*

$$ \int^{b}_{a}f(s)g(s)\,ds=g(a) \int^{\xi }_{a}f(s)\,ds+g(b) \int^{b}_{\xi }f(s)\,ds. $$

We let

$$ I_{1}(t)=\min_{x\in \mathbb{S}}\bigl(u_{x}(t,x) \bigr),\quad\quad I_{2}(t)= \max_{x\in \mathbb{S}} \bigl(u_{x}(t,x)\bigr), $$

then a new wave-breaking result is collected in the following theorem.

### Theorem 4.1

*Let* $z_{0}=(u_{0},\rho_{0})\in H^{s}( \mathbb{S})\times H^{s-1}(\mathbb{S})$ *with* $s\geq 2$, *and let* *T* *be the maximal existence time of the corresponding solution to system* (1) *with the initial data* $z_{0}$. *Assume that* $I_{1}(0)+I_{2}(0) \geq -\frac{98}{3}\mu_{0}+2\mu_{1}$, $\mu_{0}<0$, *and* $\sigma \geq 1$.

*If there are some* $x_{1}, x_{2}\in \mathbb{S}$ *such that*

38 $$\begin{aligned} \rho_{0}(x_{1})=0,\quad\quad u_{0,x}(x_{1})= \inf_{x\in \mathbb{S}}u_{0,x}(x) \end{aligned}$$

*and*

39 $$\begin{aligned} \rho_{0}(x_{2})=0,\quad\quad u_{0,x}(x_{2})= \sup_{x\in \mathbb{S}}u_{0,x}(x) , \end{aligned}$$

*then the solution of system* (1) *blows up in finite time*.

### Proof

By Theorem 2.1, we need only to prove this theorem for $s\geq 3$. According to Lemma 3.1, there exists $\xi (t)\in \mathbb{S}$ such that

40 $$\begin{aligned} I_{1}(t)=u_{x}\bigl(t,\xi (t)\bigr)=\inf _{x\in \mathbb{S}}u_{x}(t,x), \quad t \in [0,T). \end{aligned}$$

Since $q(t,\cdot )$ defined by (14) is a diffeomorphism of the circle for any $t\in [0,T)$, we know that there exists $x_{1}(t) \in \mathbb{S}$ such that

41 $$\begin{aligned} q\bigl(t,x_{1}(t)\bigr)=\xi (t), \quad t\in [0,T). \end{aligned}$$

Then (38) and (40) imply that

42 $$\begin{aligned} I_{1}(0)=u_{x}\bigl(0,\xi (0)\bigr)=\inf _{x\in \mathbb{S}}u_{0,x}(x)=u_{0,x}(x _{1}), \quad t\in [0,T). \end{aligned}$$

Therefore we can choose $\xi (0)=x_{1}$ and

43 $$\begin{aligned} \rho_{0}\bigl(\xi (0)\bigr)=\rho_{0}(x_{1})=0, \quad t\in [0,T). \end{aligned}$$

Using Lemma 3.3, we have

44 $$\begin{aligned} \rho \bigl(t,-q\bigl(t,x_{1}(t)\bigr)\bigr)=\rho \bigl(t,-\xi (t) \bigr)=0, \quad \forall t\in [0,T). \end{aligned}$$

On the other hand, due to $\sup_{x\in \mathbb{S}}(v_{x}(t,x))=- \inf_{x\in \mathbb{S}}(-v_{x}(t,x))$, we similarly define

45 $$\begin{aligned} I_{2}(t)=u_{x}\bigl(t,\eta (t)\bigr)=\sup _{x\in \mathbb{S}}u_{x}(t,x), \quad t \in [0,T). \end{aligned}$$

There exists $x_{2}(t)\in \mathbb{S}$ such that

46 $$\begin{aligned} q\bigl(t,x_{2}(t)\bigr)=\eta (t), \quad t\in [0,T). \end{aligned}$$

Moreover, we have

47 $$\begin{aligned} \rho \bigl(t,-q\bigl(t,x_{2}(t)\bigr)\bigr)=\rho \bigl(t,-\eta (t) \bigr)=0, \quad \forall t\in [0,T). \end{aligned}$$

Differentiating the first equation of system (7) with respect to *x* yields

48 $$\begin{aligned} u_{tx}-\sigma u_{x}^{2}-(\sigma u+ \gamma_{1})u_{xx}=A^{-1}\partial ^{2}_{x}\biggl(2\mu_{0}u+\frac{\sigma }{2}u^{2}_{x}+ \frac{1}{2}\rho^{2}\biggr) . \end{aligned}$$

Using (10), we get

49 $$\begin{aligned} u_{tx}-(\sigma u+\gamma_{1})u_{xx}= \frac{\sigma }{2}u_{x}^{2}+2\mu _{0}A^{-1} \partial^{2}_{x}u-\frac{1}{2}\rho^{2} + \int^{1}_{0}\frac{ \sigma }{2}u_{x}^{2}+ \frac{1}{2}\rho^{2}\,dx . \end{aligned}$$

Recalling the definitions of $I_{i}$ ($i=1,2$), we get

50 $$\begin{aligned} \frac{dI_{1}}{dt}&=\frac{\sigma }{2}I^{2}_{1}+2 \mu_{0}\partial^{2} _{x}A^{-1}u+ \int^{1}_{0}\biggl(\frac{\sigma }{2}u^{2}_{x}+ \frac{1}{2}\rho ^{2}\biggr)\,dx \\ & =\frac{\sigma }{2}I^{2}_{1}+2\mu_{0} \int^{1}_{0}g(y)u_{xx}\bigl(t,\xi (t)-y \bigr)\,dy+ \int^{1}_{0}\biggl(\frac{\sigma }{2}u^{2}_{x}+ \frac{1}{2}\rho^{2}\biggr)\,dx \end{aligned}$$

and

51 $$\begin{aligned} \frac{dI_{2}}{dt}&=\frac{\sigma }{2}I^{2}_{2}+2 \mu_{0}\partial^{2} _{x}A^{-1}u+ \int^{1}_{0}\biggl(\frac{\sigma }{2}u^{2}_{x}+ \frac{1}{2}\rho ^{2}\biggr)\,dx \\ &=\frac{\sigma }{2}I^{2}_{2}+2\mu_{0} \int^{1}_{0}g(y)u_{xx}\bigl(t,\eta (t)-y \bigr)\,dy+ \int^{1}_{0}\biggl(\frac{\sigma }{2}u^{2}_{x}+ \frac{1}{2}\rho^{2}\biggr)\,dx . \end{aligned}$$

We notice that $g(x)=\frac{1}{2}(x-\frac{1}{2})^{2}+\frac{23}{24}$ is continuous on $\mathbb{S}$, decreasing on $[0,\frac{1}{2}]$, and increasing on $[\frac{1}{2},1]$. Therefore, we have

52 $$\begin{aligned} & \biggl\vert \int^{1}_{0}g(y)u_{xx}\bigl(t,\xi (t)-y \bigr)\,dy \biggr\vert \\ &\quad = \biggl\vert \int^{\frac{1}{2}}_{0}g(y)u_{xx}\bigl(t,\xi (t)-y \bigr)\,dy \biggr\vert + \biggl\vert \int ^{1}_{\frac{1}{2}}g(y)u_{xx}\bigl(t,\xi (t)-y \bigr)\,dy \biggr\vert . \end{aligned}$$

From equality (11), we deduce

53 $$\begin{aligned} & \biggl\vert \int^{\frac{1}{2}}_{0}g(y)u_{xx}\bigl(t,\xi (t)-y \bigr)\,dy \biggr\vert \\ &\quad \leq \biggl\vert g(0) \int^{\zeta }_{0}u_{xx}\bigl(t,\xi (t)-y\bigr) \,dy \biggr\vert + \biggl\vert g\biggl( \frac{1}{2}\biggr) \int^{\frac{1}{2}}_{\zeta }u_{xx}\bigl(t,\xi (t)-y\bigr) \,dy \biggr\vert \\ &\quad =\frac{13}{12} \bigl\vert u_{x}\bigl(t,\xi (t) \bigr)-u_{x}\bigl(t,\xi (t)-\zeta \bigr) \bigr\vert + \frac{23}{24} \biggl\vert u_{x}\biggl(t,\xi (t)- \frac{1}{2}\biggr)-u_{x}\bigl(t,\xi (t)-\zeta \bigr) \biggr\vert \\ &\quad \leq \frac{49}{24}\bigl(I_{2}(t)-I_{1}(t)\bigr) . \end{aligned}$$

In an analogous way, we get

54 $$\begin{aligned} \biggl\vert \int^{1}_{\frac{1}{2}}g(y)u_{xx}\bigl(t,\xi (t)-y \bigr)\,dy \biggr\vert \leq \frac{49}{24}\bigl(I_{2}(t)-I_{1}(t) \bigr) . \end{aligned}$$

Thus, by (53) and (54), it implies

55 $$\begin{aligned} \biggl\vert \int^{1}_{0}g(y)u_{xx}\bigl(t,\eta (t)-y \bigr)\,dy \biggr\vert \leq \frac{49}{12}\bigl(I _{2}(t)-I_{1}(t) \bigr) . \end{aligned}$$

If $\mu_{0}\leq 0$ and $\sigma \geq 1$, from (55), (50), and (51) we deduce that, for *a.e.* $t\in (0,T)$,

56 $$\begin{aligned} \frac{dI_{1}}{dt} \geq \frac{\sigma }{2}I^{2}_{1}+ \frac{49}{6}\mu_{0}(I _{2}-I_{1})+ \frac{1}{2}\mu^{2}_{1} \end{aligned}$$

and

57 $$\begin{aligned} \frac{dI_{2}}{dt} &\geq \frac{\sigma }{2}I^{2}_{2}+ \frac{49}{6}\mu _{0}(I_{2}-I_{1})+ \frac{1}{2}\mu^{2}_{1} \\ & =\frac{\sigma }{2}I^{2}_{2}-\frac{49}{6} \mu_{0}(I_{2}+I_{1})+ \frac{49}{3} \mu_{0}I_{2}+\frac{1}{2}\mu^{2}_{1} . \end{aligned}$$

Summing up (56) and (57) results in

58 $$\begin{aligned} \frac{d(I_{1}+I_{2})}{dt} &\geq \frac{\sigma }{2}\bigl(I^{2}_{1}+I^{2} _{2}\bigr)+\frac{49}{3}\mu_{0}(I_{2}-I_{1})+ \mu^{2}_{1} \\ & =\frac{\sigma }{2}\bigl(I^{2}_{1}+I^{2}_{2} \bigr)+\frac{49}{3}\mu_{0}(I_{2}+I _{1})- \frac{98}{3}\mu_{0}I_{1}+\mu^{2}_{1} . \end{aligned}$$

From the assumption of Theorem 4.1 $I_{0}(0)+I_{2}(0)\geq - \frac{98}{3}\mu_{0}+2\mu_{1}$, we now claim that, for all $t\in T$,

59 $$\begin{aligned} (I_{1}+I_{2}) (t)\geq -\frac{98}{3} \mu_{0}+2\mu_{1} . \end{aligned}$$

Let $I(t)=(I_{1}+I_{2})(t)+\frac{98}{3}\mu_{0}-2\mu_{1}$. Then we claim that $I(t)\geq 0$. It is observed that $I(t)$ is continuous on $[0,T)$. Assume that $I(t)\geq 0$ is not valid, then there is $t_{0}\in (0,T)$ such that $I(t_{0})<0$. Let $t_{1}=\max \{t< t_{0}: I(t)=0 \}$. Then $I(t_{1})=0$ and $I'(t_{1})<0$, namely

60 $$\begin{aligned} (I_{1}+I_{2}) (t_{1})=-\frac{98}{3} \mu_{0}+2\mu_{1} \end{aligned}$$

and

61 $$\begin{aligned} I'(t)=\bigl(I'_{1}+I'_{2} \bigr) (t_{1})< 0 . \end{aligned}$$

Due to

62 $$\begin{aligned} I_{2}(t_{1})\geq \frac{1}{2}(I_{1}+I_{2}) (t_{1})=-\frac{98}{6}\mu_{0}+ \mu_{1} \end{aligned}$$

and

63 $$\begin{aligned} I_{1}(t_{1})=-\frac{98}{3}\mu_{0}+2 \mu_{1}-I_{2}(t_{1}) . \end{aligned}$$

Thus, we get

64 $$\begin{aligned} I'(t_{1})&=I'_{1}(t_{1})+I'_{2}(t_{1}) \\ & \geq \frac{\sigma }{2}\bigl(I^{2}_{1}+I^{2}_{2} \bigr)-\frac{49}{3}\mu_{0}(I_{2}+I _{1})+ \frac{98}{3}\mu_{0}I_{2}+\mu^{2}_{1} \\ & =\frac{\sigma }{2}I^{2}_{2}+\frac{\sigma }{2}\biggl(- \frac{98}{3}\mu_{0}+2 \mu_{1}-I_{2}(t_{1}) \biggr)^{2}-\frac{49}{3}\mu_{0}\biggl(- \frac{98}{3}\mu_{0}+2 \mu_{1}\biggr) \\ & \quad {}+\frac{98}{3}\mu_{0}I_{2}(t_{1})+ \mu^{2}_{1} \\ & =\sigma I^{2}_{2}-\sigma \biggl(-\frac{98}{3} \mu_{0}+2\mu_{1}\biggr)I_{2}+ \frac{98}{3} \mu_{0}I_{2}+\frac{\sigma }{2}\biggl(-\frac{98}{3} \mu_{0}+2\mu _{1}\biggr)^{2} \\ & \quad {}-\frac{49}{3}\mu_{0}\biggl(-\frac{98}{3} \mu_{0}+2\mu_{1}\biggr)+\mu^{2}_{1} \\ & =\sigma \biggl[I_{2}-\frac{\sigma (-\frac{98}{3}\mu_{0}+2\mu_{1})- \frac{98}{3}\mu_{0}}{2\sigma }\biggr]^{2} - \frac{[\sigma (-\frac{98}{3}\mu _{0}+2\mu_{1})-\frac{98}{3}\mu_{0}]^{2}}{4\sigma } \\ & \quad {} +\frac{\sigma }{2}\biggl(-\frac{98}{3}\mu_{0}+2 \mu_{1}\biggr)^{2} - \frac{49}{3}\mu_{0} \biggl(-\frac{98}{3}\mu_{0}+2\mu_{1}\biggr)+ \mu^{2}_{1} \\ & =\sigma \biggl[I_{2}-\frac{\sigma (-\frac{98}{3}\mu_{0}+2\mu_{1})- \frac{98}{3}\mu_{0}}{2\sigma }\biggr]^{2}+ \frac{\sigma }{4}\biggl(-\frac{98}{3}\mu _{0}+2 \mu_{1}\biggr)^{2} \\ & \quad {} -\frac{1}{4\sigma }\biggl(\frac{98}{3}\mu_{0} \biggr)^{2}+\mu^{2}_{1} \\ & >0, \end{aligned}$$

which gives rise to a contradiction with (61). Therefore, (59) is true.

65 $$\begin{aligned} &\frac{d(I_{2}(t)+\frac{49}{3\sigma }\mu_{0})}{dt} \\ &\quad =\frac{dI_{2}}{dt} \\ &\quad \geq \frac{\sigma }{2}I^{2}_{2}-\frac{49}{6} \mu_{0}(I_{2}+I_{1})+ \frac{49}{3} \mu_{0}I_{2}+\frac{1}{2}\mu^{2}_{1} \\ &\quad \geq \frac{\sigma }{2}I^{2}_{2}-\frac{49}{6} \mu_{0}\biggl(-\frac{98}{3}\mu _{0}+2 \mu_{1}\biggr)+\frac{49}{3}\mu_{0}I_{2}+ \frac{1}{2}\mu^{2}_{1} \\ &\quad =\frac{\sigma }{2}\biggl(I_{2}(t)+\frac{49}{3\sigma } \mu_{0}\biggr)^{2}-\frac{1}{2 \sigma }\biggl( \frac{49}{3}\mu_{0}\biggr)^{2}+\biggl( \frac{49}{3}\mu_{0}\biggr)^{2} - \frac{49}{3} \mu_{0}\mu_{1}+\frac{1}{2}\mu^{2}_{1} \\ &\quad \geq \frac{\sigma }{2}\biggl(I_{2}(t)+\frac{49}{3\sigma } \mu_{0}\biggr)^{2}. \end{aligned}$$

Since $I_{2}(t)$ is locally Lipschitz on $(0,T)$, we have that $\frac{1}{(I_{2}(t)+\frac{49}{3\sigma }\mu_{0})}$ is also locally Lipschitz on $(0,T)$, then $\frac{1}{(I_{2}(t)+\frac{49}{3\sigma }\mu _{0})}$ is absolutely continuous on $(0,T)$.

Solving (65), we obtain

66 $$\begin{aligned} \frac{1}{(I_{2}(t)+\frac{49}{3\sigma }\mu_{0})}\leq \frac{1}{(I_{2}(0)+\frac{49}{3 \sigma }\mu_{0})}-\frac{\sigma }{2}t , \end{aligned}$$

which leads us to

67 $$\begin{aligned} I_{2}(t)>\frac{(I_{2}(0)+\frac{49}{3\sigma }\mu_{0})}{1-\frac{\sigma }{2}t(I_{2}(0)+\frac{49}{3\sigma }\mu_{0})}-\frac{49}{3\sigma }\mu _{0} . \end{aligned}$$

The above inequality implies that $I_{2}(t)\rightarrow +\infty $ as $t\rightarrow \frac{2}{\sigma (I_{2}(0)+\frac{49}{3\sigma }\mu_{0})}$. Applying Theorem 3.2, we complete the proof of Theorem 4.1. □

### Remark 1

If we let $\rho_{0}(-x)=0$, then from Lemma 3.3 we can obtain $\rho (t,-x)=0$ easily. Then system (1) is degenerated into *μ*-version Camassa–Holm equation under $\gamma_{1}=0$. For the blow-up results related to *μ*-version Camassa–Holm equation, the reader is referred to [6] and the references therein.

### Remark 2

It is worthwhile to mention that comparing with the results in [2], our blow-up results are new and quite different. There is twofold meaning: firstly, our blow-up criteria and the proof of them are different from the ones in [2]. Then, our blow-up phenomena (see Theorem 4.1) are also different from the ones in [2], because the conditions of Theorem 4.1 in our paper are different from the ones [2]. When $\rho_{0}(-x)=0$, system (1) is degenerated into *μ*-version Camassa–Holm equation essentially. So the blow-up phenomena in [2] belong to *μ*-version Camassa–Holm equation.
